# Cytokine TGFβ Gene Polymorphism in Asthma: TGF-Related SNP Analysis Enhances the Prediction of Disease Diagnosis (A Case-Control Study With Multivariable Data-Mining Model Development)

**DOI:** 10.3389/fimmu.2022.746360

**Published:** 2022-06-14

**Authors:** Michał Panek, Konrad Stawiski, Marcin Kaszkowiak, Piotr Kuna

**Affiliations:** ^1^ Department of Internal Medicine, Asthma and Allergy of The Medical University of Lodz, Medical University of Lodz, Lodz, Poland; ^2^ Department of Biostatistics and Translational Medicine of The Medical University of Lodz, Medical University of Lodz, Lodz, Poland

**Keywords:** TGF — transforming growth factor, asthma, inflammation, prediction, development

## Abstract

**Introduction:**

TGF-β and its receptors play a crucial role in asthma pathogenesis and bronchial remodeling in the course of the disease. TGF-β1, TGF-β2, and TGF-β3 isoforms are responsible for chronic inflammation, bronchial hyperreactivity, myofibroblast activation, fibrosis, bronchial remodeling, and change the expression of approximately 1000 genes in asthma. TGF-β SNPs are associated with the elevated plasma level of TGF-β1, an increased level of total IgE, and an increased risk of remodeling of bronchi.

**Methods:**

The analysis of selected TGF-β1, TGF-β2, TGF-β3-related single-nucleotide polymorphisms (SNP) was conducted on 652 DNA samples with an application of the MassARRAY^®^ using the mass spectrometry (MALDI-TOF MS). Dataset was randomly split into training (80%) and validation sets (20%). For both asthma diagnosis and severity prediction, the C5.0 modelling with hyperparameter optimization was conducted on: clinical and SNP data (Clinical+TGF), only clinical (OnlyClinical) and minimum redundancy feature selection set (MRMR). Area under ROC (AUCROC) curves were compared using DeLong’s test.

**Results:**

Minor allele carriers (MACs) in SNP rs2009112 [OR=1.85 (95%CI:1.11-3.1), p=0.016], rs2796821 [OR=1.72 (95%CI:1.1-2.69), p=0.017] and rs2796822 [OR=1.71 (95%CI:1.07-2.71), p=0.022] demonstrated an increased odds of severe asthma. Clinical+TGF model presented better diagnostic potential than OnlyClinical model in both training (p=0.0009) and validation (AUCROC=0.87 vs. 0.80,p=0.0052). At the same time, the MRMR model was not worse than the Clinical+TGF model (p=0.3607 on the training set, p=0.1590 on the validation set), while it was better in comparison with the Only Clinical model (p=0.0010 on the training set, p=0.0235 on validation set, AUCROC=0.85 vs. 0.87). On validation set Clinical+TGF model allowed for asthma diagnosis prediction with 88.4% sensitivity and 73.8% specificity.

**Discussion:**

Derived predictive models suggest the analysis of selected SNPs in TGF-β genes in combination with clinical factors could predict asthma diagnosis with high sensitivity and specificity, however, the benefit of SNP analysis in severity prediction was not shown.

## Introduction

### Background/Rationale

The latest concept of chronic airway inflammation in asthma implies the existence of a complex interaction between the epithelium, innate lymphoid cells (ILCs), lymphocytes and, finally, effector cells. Current advances in basic sciences have allowed researchers to discover three basic forms of responses of airway epithelium to allergic and non-allergic factors leading to its damage. Such a division results from the discovery of various types of ILCs, cytokine profiles and responses of the effector cells (1, 2). Type 1 immunity consists of T-bet(+) IFN-γ-producing group 1 ILCs (ILC1) and natural killer cells, CD8(+) cytotoxic T cells (Tc1), and CD4(+) Th1 cells and forms a mechanism protecting against viral infections. Type 2 immunity is composed of GATA-3(+) ILC2s, Tc2 cells, and Th2 cells producing IL-4, IL-5, and IL-13 which activate mast cells, B lymphocytes, basophils, and eosinophils and are responsible for anti-allergic and anti-parasitic reactions. Type 3 immunity is regulated by the retinoic acid-related orphan receptor γt(+) ILC3s, Tc17 cells, and Th17 cells producing IL-17, IL-22, or both, and mediates antifungal and antibacterial reactions. On the other hand, types 1 and 3 determine the development of autoimmune diseases (non-allergic diseases), while type 2 is responsible for the development of allergic diseases (2).

It is the epithelium/Th2/ILC2 system that determines the lack of control of asthma symptoms, progression of the disease and development of its complications. Eosinophils can induce EMT (Epithelial-Mesenchymal Transition) in airway epithelial cells *via* increased production of the transforming growth factor (TGFβ), which is the main and most important molecular and cellular mechanism causing airway remodeling. This data has been confirmed by the latest experimental research (3–5). Experimental murine airway remodeling models have proven that blocking TGF-β mediated inflammation by targeting Smad proteins, c-Jun N-terminal kinase and phosphoinositide 3-kinase signaling pathways decreases bronchial fibrosis. Undoubtedly, the above proteins are responsible for chronic inflammation, bronchial hyperreactivity, myofibroblast activation, fibrosis, bronchial remodeling, and they change the expression of approximately 1000 genes in asthma, especially those of MMPs, PAI-1, CTGF, MCP-1, IL-6, TGF-β, TSP-1, TGFR-1/2, fibronectine, proteoglycans, as well as type I and III collagen (3, 4, 6).

The TGFβ (1-3 isoforms, are small, 25 kDa secreted homodimeric signaling proteins) and especially TGFβ1 superfamily is responsible for immunosuppresion of T and B lymphocytes as well as NK cells, chemotaxis of macrophages and fibroblasts, stimulation of proliferation, an increase in fibroblast synthesis, stimulation of synthesis of fibronectin, proteoglycans, and type I and III collagen, eosinophil chemotaxis after allergen exposure, MAP kinase phosphorylation, increase in bronchial myocyte proliferation, inhibition of collagenase and matrix metalloproteinase gene expression, inhibition of MHC class II antigen expression and inhibition of surfactant synthesis by type II pneumocytes (6, 7). On the other hand, hyperactivity of the TGFβ-Smad (a family of proteins similar to the gene products of the Drosophila gene ‘mothers against decapentaplegic’ (Mad) and the C. elegans gene Sma) signaling pathway underlies many human disorders, such as excess deposition of the extracellular matrix, fibrotic disorders, and progressive cancers (6–8).

Expression of isoforms of TGFβ 1 - 3 cytokines is influenced by tagging single nucleotide polymorphisms (SNPs) in the TGFβ1, TGFβ2 and TGFβ3 genes, which may be associated with asthma and other diseases. TGFβ1- TGFβ3 gene regulation and expression levels are affected by presence of SNP in the *locus* (8–13).

Numerous studies conducted on diverse populations have shown that genetic factors largely contribute to variability in the pulmonary function and to familial aggregation of asthmatic patients.

We detected base substitutions as single-stranded conformational polymorphisms. We screened each polymorphism by a case-control analysis in order to find association with allergy and asthma using our data base containing 237 atopic asthmatic and 268 non-asthmatic families. **Table 1** presents analyzed polymorphisms in the TGFβ1, TGFβ2 and TGFβ3 genes. Polymorphism rs8179181 in the TGFβ1 gene is associated with an increased risk of childhood asthma and atopy. It is associated with a more severe course of the disease and increased levels of TGFβ1 mRNA (8). rs4803455 correlates with the risk of the disease. Moreover, it worsens the lung function and causes airway remodeling in asthma (9). rs1800469 in the TGF-β1 promoter has been found to be related to an elevated plasma level of TGF-β1, an elevated level of total IgE and an increased risk of remodeling bronchi, as well as the development of asthma (10–13). rs11083616 is associated with bronchial obturation as well as with airway wall phenotypes - airway wall thickness. It is a significant risk of obturatory diseases (14). It has not really confirmed that rs8109627 in the TGFβ1 gene contributes to an increased risk of asthma. The role of tagging polymorphisms in the TGFβ2 gene (rs10495098, rs17047703, rs17558745, rs2799085, rs2009112, rs10482751, rs2027567, rs10779329, rs2796821, rs2796822, rs4846479, rs2798631, rs10863399) as well as in the TGFβ3 gene (rs4903359, rs3917187, rs2284792, rs2268626) has not been confirmed yet. Nevertheless, it should be stressed that asthma phenotypic differences that result from altered expression due to SNPs are sometimes inconsistent and disease association studies are often ambiguous (15).

**Table 1:** Characteristics of tagging SNP polymorphisms in TGFβ1, TGFβ2 and TGFβ3 genes on the basis of the dbSNP database of the National Center for Biotechnology Information, U.S. National Library of Medicine (8600 Rockville Pike, Bethesda MD, 20894 USA).

Gene	SNP ID	Variant type	Alleles	Chromosome	MAF (1000Genomes)	Functional Consequence
TGF-β1	rs8109627	SNV	T>C	19:41317081	C=0.3696/1851	intron variant
	rs8179181	SNV	G>A,C,T	19:41332301	A=0.0761/381	intron variant
	rs4803455	SNV	C>A	19:41345604	A=0.4772/2390	intron variant
	rs1800469	SNV	A>G	19:41354391	A=0.3680/1843	upstream transcript variant
	rs11083616	SNV	G>A	19:41359738	G=0.4443/2225	intron variant
TGF-β2	rs10495098	SNV	G>A,T	1:218342968	T=0.4585/2296	no data available in the dbSNP NCBI
	rs17047703	SNV	C>A	1:218352246	A=0.2482/1243	intron variant
	rs17558745	SNV	C>T	1:218375179	T=0.2304/1154	intron variant
	rs2799085	SNV	A>C,T	1:218379113	A=0.4393/2200	intron variant
	rs2009112	SNV	C>T	1:218380187	T=0.1865/934	intron variant
	rs10482751	SNV	T>C	1:218382955	C=0.4367/2187	intron variant
	rs2027567	SNV	G>A	1:218385246	G=0.4391/2199	intron variant
	rs10779329	SNV	T>C	1:218400399	T=0.4972/2490	intron variant
	rs2796821	SNV	C>T	1:218412479	T=0.3021/1513	intron variant
	rs2796822	SNV	A>G	1:218412790	A=0.3097/1551	intron variant
	rs4846479	SNV	G>T	1:218425068	T=0.4183/2095	intron variant
	rs2798631	SNV	A>G	1:218438536	A=0.2861/1433	intron variant
	rs10863399	SNV	A>C	1:218453334	C=0.2073/1038	no data available in the dbSNP NCBI
TGF-β3	rs4903359	SNV	G>A,C	14:75944394	G=0.2280/1142	intron variant
	rs3917187	SNV	T>C	14:75965793	T=0.4093/2050	intron variant
	rs2284792	SNV	G>A,C	14:75977236	G=0.4028/2017	intron variant
	rs2268626	SNV	C>T	14:75978424	C=0.2288/1146	intron variant

SNV, Single Nucleotide Variant.

### Aims and Objectives

The aim of our study was to identify SNPs in TGF-β family potentially associated with asthma occurrence and severity, and subsequently test their predictive value. To that end, we decided to evaluate the prevalence of SNPs in TGF-β1, TGF-β2 and TGF-β3 in both asthmatic and non-asthmatic polish population. Collected data were intended to serve as a base for binary classification models.

## Methods

### Consent of the Bioethics Committee

The study was approved by the local ethics committee (Consent of Research Review Board at the Medical University of Lodz, Poland, No RNN/133/09/KE). At the commencement of the study, participants were invited to take part voluntarily. Before enrollment, a written informed consent was obtained from each patient.

### Variables and Subjects

Asthma diagnosis was established according to GINA (The Global Initiative For Asthma) recommendations, based on clinical asthma symptoms and a lung function test. The level of asthma severity and control was determined on the basis of GINA Report Guidelines. All the participants underwent structuralized anamnesis and clinical examination, to collect details on factors such as: gender, obesity, tobacco smoking, duration of bronchial asthma, allergy to house dust mites, animal fur, mould spores, cockroaches allergens, hypersensitivity to non-steroid anti-inflammatory drugs (NSAIDs), etc., in order to determine their role in the development of resistance to glucocorticoids, as well as to establish genetic predisposition (obtained from medical records of particular patients). If results of spirometry or allergological tests were unavailable, such examinations were additionally performed during the recruitment visit. Subjects suffering from clinically significant exacerbations, using drugs which might induce resistance to glucocorticoids (such as rifampicin, phenobarbital, phenytoin, effedrine), subjects with signs of viral infections, either generalized, or affecting the respiratory tract, as well as subjects failing to comply with the doctor’s recommendations, were excluded from the patient group. The control arm included a group of healthy adults, who met the following criteria: no history or symptoms of either bronchial asthma or other pulmonary diseases, no history or symptoms of allergy, no history or symptoms of atopic dermatitis, no history, or signs of hypersensitivity to aspirin, negative results of skin tests for 12 common allergens, no first-degree relatives with bronchial asthma or atopic disorders. Spirometry tests were conducted in the Outpatient Department according to ERS (European Respiratory Society)/ATS (American Thoracic Society) standards, whereas allergological tests were performed according to EAACI (European Academy of Allergy and Clinical Immunology) guidelines (10–13, 16, 17).

The whole study group consisted of 652 individuals whose mean age was 47.4 ± 15.9 years. Detailed characteristics of the patients were presented in **Table 2**.

**Table 2:** Clinical characteristics of the recruited cohort and presentation of spirometric characteristics of studied groups.

Characteristics of the studied groups	Asthmatic Patients (n = 345)	Healthy Controls (n = 307)	p-value
Age [years]	48.6 ± 15.4	46.0 ± 16.3	0.04
Sex	Females: 222 Males: 123	Females: 197 Males: 110	0.96
Height [meters]	1.67 ± 0.10	1.68 ± 0.11	0.72
Weight [kilograms]	75.59 ± 15.30	73.29 ± 16.92	0.07
BMI [kilograms/m^2^]	26.95 ± 4.80	25.73 ± 5.05	<0.01
Allergy	None: 148 Seasonal: 40 Year-round: 58 Both: 99	None: 268 Seasonal: 8 Year-round: 6 Both: 25	<0.001
FEV1 (%) pred.	75.81 ± 21.37	95.83 ± 19.95	<0.001
FVC (%) pred.	93.52 ± 18.78	101.63 ± 17.69	<0.001
FEV1/FVC (%) pred.	83.25 ± 14.71	95.88 ± 10.05	<0.001
Smoking pack years	5.82 ± 10.98	5.68 ± 11.71	0.88

FEV1 (forced expiratory volume in 1 second) expressed in %, FEV1% (A/N% - percentage ratio of the measured to expected value) expressed as per cent of the expected value; FVC (forced vital capacity) expressed in %, FVC% (A/N% - percentage ratio of the measured to expected value) expressed as per cent of the expected value; FEV1% FVC index (FEV1 to FVC ratio - forced vital capacity) expressed in %. pred., predicted.

### Genomic DNA Extraction and SNPs Analysis (MassARRAY ^®^ System)

The DNA was isolated from peripheral blood leukocyte fraction using QIAamp DNA Blood Mini Kit (QIAGEN Inc.) according to protocol (12, 13, 17–19). DNA impurity, defined as the A260/A280 absorbance ratio, ranged from 1.7 to 2.0.

TGF-β1, TGF-β2, TGF-β3 polymorphism detection was performed using MassARRAY^®^ system [(MassARRAY Analyzer 4; The MassARRAY^®^ System by Agena Bioscience^®^) Bionanopark, Lodz, Poland], with procedure and preprocessing steps performed according to the standard protocol.

### Statistical Methods

The statistical analysis was performed with an application of Welch two-sample t-test and Pearson’s Chi-squared test (with Yates’ continuity correction if appropriate) in intragroup association testing. Unadjusted Chi-Square test statistic was also used in pairwise linkage disequilibrium analysis prior to the modelling. Standard r-squared (r2) and p-values were calculated for each pair.

Logistic regression analysis was performed to derive odds ratios with their confidence intervals in univariable analysis. In the first step, the analysis was performed for minor allele carriers (MACs) i.e. presence of at least one minor allele (so called recessive model). In this analysis, the lack of a minor allele was considered as reference. The second step of the analysis included testing the association of particular genotypes (i.e. additive model) with asthma diagnosis and severity, with the most common genotype considered as a reference. The goodness of fit was assessed using the likelihood-ratio test (LR-test). Just before the analysis, as missing data constituted only the 2% (n=723/35860) of the overall dataset, multiple imputation using chained equations was performed. Predictive mean matching was performed with a maximum of 500 iterations.

In order to create the clinically useful multivariable models, the data-mining procedures followed the gold-standards of predictive model development. Since both asthma occurrence and asthma severity were variables of interest, binary classification models were created for asthma diagnosis (asthma vs healthy) and asthma severity (mild vs severe; using only data asthmatic patients), respectively. Both scenarios were executed independently, with identical steps.

Firstly, the dataset was randomly divided (with stratification) into training and validation set in 80%:20% ratio. To answer whether addition of SNP-related data adds to discriminatory power of models, we developed models for 3 scenarios (1): jointly clinical data and TGF-related SNPs (scenario further referred to as “TGF+clinical”) (2), only clinical data (scenario further referred to as “clinical”) (3), selected features from clinical data and TGF-related SNPs using minimum redundancy feature selection (scenario further referred to as “MRMR”). Predictive models were developed using Quinlan’s C5.0 algorithm with hyperparameter optimization (including 500 iterations of random search). As decision trees employ own build-it feature selection and pruning, no additional feature selection was performed. To counteract possible overfitting, the best set of hyperparameters was selected based on the accuracy of metrics derived from 10-fold cross-validation performed on the training set, thus the validation set had no impact on selection of best hyperparameters.

The Quinlan’s C5.0 algorithm extends the C4.5 classification algorithm and can produce decision trees or collections of rules. Both of those can be further boosted, creating ensemble models. Information gain (entropy) is used as its splitting criteria, while C5.0 pruning technique adopts the binomial confidence limit method. All of those lead to detection of far more complex patterns than frequently used logistic regression.

**Figure 1:**
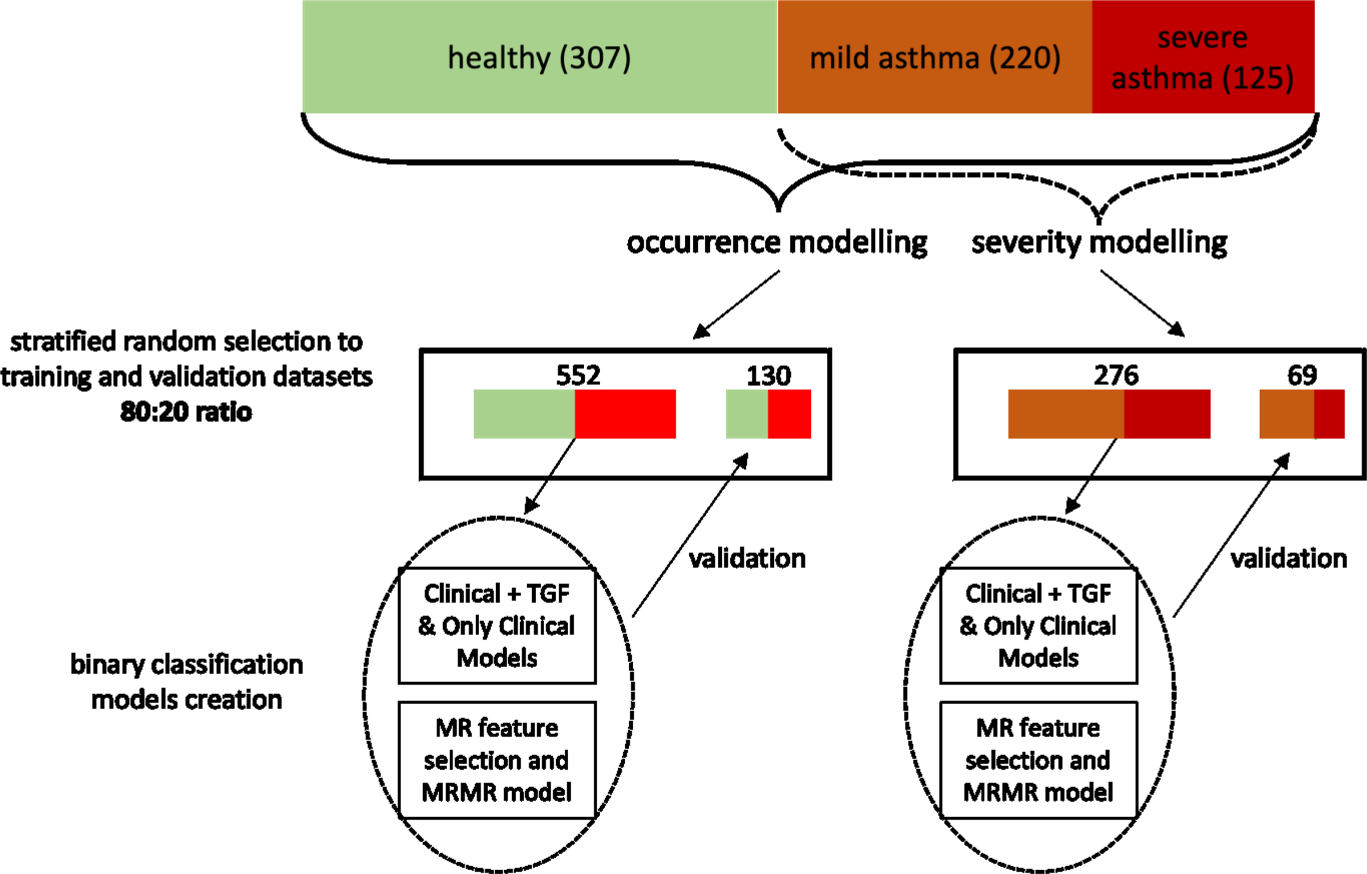
The flowchart showing the pipeline of model development and validation. I confirm that all methods were performed in accordance with the relevant guidelines and regulations.

The best models were finally validated on hold-out validation set. To avoid bias, the minimum redundancy feature selection (MRMR) was performed after dataset splitting. (**Figure 1**) DeLong’s test for two correlated ROC curves (receiver operating characteristic curves) was used to compare predictive abilities between sets and scenarios. The DeLong method was also applied in calculations of 95% confidence intervals (95%CI) for the area under the ROC curves (AUC ROC) (20, 21).

The analysis was performed utilizing R programming languages (version 3.6.1) with the following crucial packages: caret (version 6.0-84), mRMRe (version 2.0.9) and C50 (version 0.1.2). Final *caret* models were extracted in RDS format and placed as **Supplementary Files** for reproducibility and further validations. As all comparisons were preplanned, no multiple comparison correction was applied. The analysis code was published in GitHub repository: https://github.com/kstawiski/Panek_TGF. Additional data may be provided upon readers’ requests (20, 21).

## Results

### Participants

A comparative analysis of biometric parameters revealed differences with asthmatic patients and healthy controls. Detailed characteristics of the patients were presented in **Table 2**. As it was assumed, differences in sex, height, and weight as well as in smoking pack-years were not statistically significant. However, we have noticed that healthy controls were slightly younger and had a lower BMI. The difference in BMI was not noticed between patients with severe and non-severe asthma (**Table 3**).

**Table 3:** Clinical characteristics of asthmatic patients regarding asthma severity, * - uncertain cases were excluded. pred. - predicted.

Parameter	Non-severe asthma (n=220)	Severe asthma (n=125)	P-value
Age [years]	47.4 ± 15.6	50.7 ± 14.3	0.05
Sex	Females: 142 Males: 78	Females: 80 Males: 45	0.92
Height [meters]	1.68 ± 0.10	1.67 ± 0.09	0.37
Weight [kilograms]	75.46 ± 15.36	75.81 ± 15.25	0.84
BMI [kilograms/m^2^]	26.80 ± 4.74	27.22 ± 4.91	0.44
Allergy	None: 99 Seasonal: 22 Year-round: 39 Both: 60	None: 49 Seasonal: 18 Year-round: 19 Both: 39	0.44
FEV1(%) pred.	83.65 ± 17.63	62.01 ± 20.41	<0.001
FVC(%) pred.	99.09 ± 15.17	83.72 ± 20.5	<0.001
FEV1/FVC(%) pred.	87.40 ± 11.89	75.96 ± 16.32	<0.001
Age of diagnosis*	<3 years: 6 3-7 years: 10 7-16 years: 21 16-40 years: 90 >40 years: 84	<3 years: 12 3-7 years: 6 7-16 years: 11 16-40 years: 55 >40 years: 39	0.08
Asthma Control Test score	19.14 ± 4.64	15.23 ± 5.55	<0.001
Multiple exacerbations	Yes: 14 No: 206	Yes: 63 No: 62	<0.001
Smoking pack years	5.41 ± 9.35	6.53 ± 13.38	0.41

**Figure 2:**
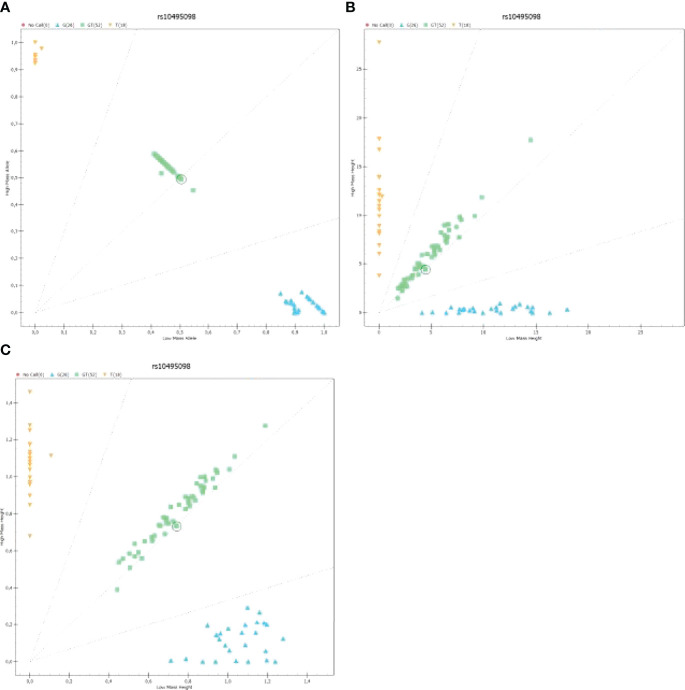
The graph presenting the distribution of homo- and heterozygotes for polymorphism rs10495098 for all samples analyzed on a 96-well plate: **(A)** The graph presenting the distribution of low-mass C homozygote (Low Mass Allele) to high-mass A homozygotes depending on the yield; **(B)** The graph presenting the heights of peaks being signals of mass spectra for homo- and heterozygotes; **(C)** The graph presenting the logarithmic value of signal intensity of mass spectra for homo- and heterozygotes [Log [Height]). .

An analysis of samples with an application of mass spectrometer MassARRAY4, the authors obtained raw results (**Supplementary Figure 1**) presented in the form of mass spectra. They were used to detect polymorphisms in the studied genes. **Figure 2** presents a distribution of homo- and heterozygotes for all analyzed samples depending on the yield (**Figure 2A**), height of the mass spectrum peak (**Figure 2B**) and common logarithm (LOG) from the height of the mass spectrum peak (**Figure 2C**).

The graphs presented in **Figure 2** show a result of an analysis of rs2009112 polymorphism for three randomly selected samples. They are image representations and output data for identification of polymorphism in the analyzed sample. **Supplementary Figure 2**

**Figure 3:**
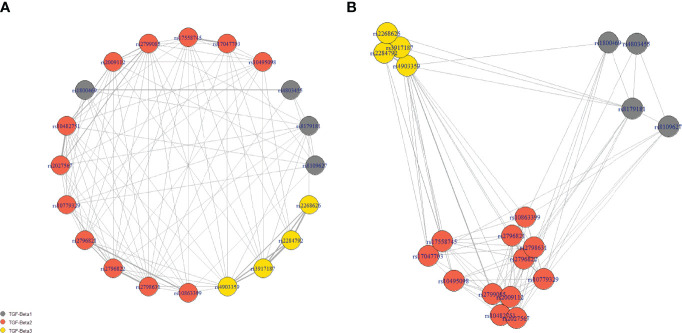
The network of statistically significant disequilibrium. Panel **(A)** presents the nodes in the circle, while the location of nodes in panel **(B)** is dependent on the strength of the association (scaled r2 values). Grey lines show significant linkage disequilibrium between SNPs in the pairwise analysis. The weight of the line is calculated based on the r2 values. The length of connection corresponded inversely to the strength of association.

Similarly to the Hardy-Weinberg principle, the co-occurrence of different SNPs should be theoretically random. However, the pairwise linkage disequilibrium analysis showed that 100 out of 190 comparisons were significantly associated. Not surprisingly, based on the r2 values, the strongest associations were found to be between SNPs from the same genes. However, the results od this analysis were rather mixed, indicating complex genetic landscape of selected SNPs. Please see the network of statistically significant disequilibrium on **Figure 3**. Information redundancy and significant associations between SNPs further supported application of feature selection and data-mining modeling with embedded feature selection.

As shown in **Table 4**, the univariable analysis has revealed a significance of minor allele carriers (MACs) of rs2009112, rs2796821, and rs2796822 regarding severe asthma development. None of the SNPs was significantly associated with the risk of asthma in the univariable analysis.

**Table 4:** Univariable analysis with odds ratios (OR) for minor allele carriers (MACs) and particular genotypes in comparison with reference (ref., most common) genotype.

Analyzed groups	Asthmatic vs. control patients		Severe vs. non-severe asthma	
Studied SNPs	OR (95%CI)	P (LR-test)	OR (95%CI)	P (LR-test)
MAC of rs8109627	1.07 (0.78,1.47)	0.657	0.78 (0.5,1.22)	0.273
MAC of rs8179181	0.89 (0.65,1.21)	0.45	0.85 (0.55,1.33)	0.476
MAC of rs4803455	0.91 (0.65,1.29)	0.612	0.68 (0.42,1.11)	0.123
MAC of rs1800469	1.09 (0.8,1.48)	0.598	1.27 (0.81,1.98)	0.295
MAC of rs10495098	1.03 (0.74,1.43)	0.88	1.02 (0.64,1.65)	0.921
MAC of rs17047703	0.95 (0.69,1.3)	0.746	0.84 (0.53,1.32)	0.442
MAC of rs17558745	1.18 (0.87,1.61)	0.287	0.67 (0.43,1.04)	0.075
MAC of rs2799085	0.97 (0.71,1.33)	0.841	0.96 (0.61,1.5)	0.852
MAC of rs2009112	1.06 (0.76,1.49)	0.729	1.85 (1.11,3.1)	0.016
MAC of rs10482751	1.02 (0.75,1.39)	0.907	1.01 (0.65,1.57)	0.956
MAC of rs2027567	1.03 (0.76,1.41)	0.836	0.83 (0.54,1.3)	0.417
MAC of rs10779329	0.83 (0.61,1.14)	0.249	0.93 (0.6,1.45)	0.746
MAC of rs2796821	1.2 (0.88,1.65)	0.255	1.72 (1.1,2.69)	0.017
MAC of rs2796822	0.96 (0.7,1.31)	0.79	1.71 (1.07,2.71)	0.022
MAC of rs2798631	0.88 (0.63,1.22)	0.437	1.47 (0.91,2.38)	0.113
MAC of rs10863399	1.1 (0.79,1.53)	0.589	1.54 (0.96,2.45)	0.072
MAC of rs4903359	0.74 (0.55,1.01)	0.06	1.04 (0.67,1.61)	0.863
MAC of rs3917187	1.15 (0.84,1.57)	0.39	0.94 (0.6,1.47)	0.79
MAC of rs2284792	1.18 (0.86,1.61)	0.298	1.01 (0.65,1.58)	0.948
MAC of rs2268626	1.02 (0.73,1.41)	0.914	1.32 (0.83,2.09)	0.24
rs8109627ref.=T/T		0.86		0.057
T/C	1.09 (0.79,1.51)		0.67 (0.41,1.08)	
C/C	0.97 (0.49,1.94)		1.99 (0.75,5.25)	
rs8179181ref.=G/G		0.728		0.665
G/A	0.88 (0.63,1.21)		0.89 (0.56,1.42)	
A/A	0.95 (0.53,1.71)		0.69 (0.29,1.66)	
rs4803455ref.=C/C		0.847		0.109
C/A	0.9 (0.62,1.3)		0.78 (0.47,1.3)	
A/A	0.95 (0.61,1.46)		0.51 (0.27,0.96)	
rs1800469ref.=G/G		0.251		0.547
G/A	1.08 (0.79,1.47)		1.26 (0.81,1.98)	
A/A	726582.93 (0,Inf)		2.02 (0.12,32.99)	
rs10495098ref.=G/G		0.214		0.895
G/T	0.93 (0.65,1.32)		1.07 (0.64,1.77)	
T/T	1.35 (0.86,2.13)		0.93 (0.5,1.74)	
rs17047703ref.=C/C		0.923		0.166
C/A	0.96 (0.69,1.34)		0.93 (0.58,1.49)	
A/A	0.87 (0.41,1.88)		0.28 (0.06,1.26)	
rs17558745ref.=C/C		0.504		0.142
C/T	1.22 (0.88,1.69)		0.71 (0.45,1.13)	
T/T	1.04 (0.57,1.9)		0.47 (0.18,1.22)	
rs2799085ref.=C/C		0.306		0.858
C/A	1.06 (0.75,1.48)		0.9959(0.6206,1.5982)	
A/A	0.74 (0.47,1.18)		0.83 (0.41,1.69)	
rs2009112ref.=C/C		0.76		0.056
C/T	1.02 (0.71,1.46)		1.83 (1.06,3.16)	
T/T	1.16 (0.76,1.77)		1.9 (1.02,3.54)	
rs10482751ref.=C/C		0.454		0.966
C/T	1.09 (0.79,1.51)		1.03 (0.65,1.63)	
T/T	0.78 (0.46,1.31)		0.93 (0.42,2.06)	
rs2027567ref.=A/A		0.866		0.667
A/G	1.06 (0.77,1.46)		0.86 (0.54,1.36)	
G/G	0.9 (0.49,1.67)		0.71 (0.28,1.81)	
rs10779329ref.=T/T		0.091		0.886
T/C	0.92 (0.67,1.28)		0.95 (0.6,1.51)	
C/C	0.53 (0.3,0.94)		0.8 (0.31,2.04)	
rs2796821ref.=C/C		0.485		0.004
C/T	1.22 (0.88,1.69)		1.93 (1.22,3.05)	
T/T	1.04 (0.46,2.32)		0.4 (0.09,1.88)	
rs2796822ref.=A/A		0.26		0.004
A/G	1.05 (0.75,1.47)		2.02 (1.25,3.28)	
G/G	0.72 (0.46,1.15)		0.87 (0.41,1.85)	
rs2798631ref.=A/A		0.483		0.284
A/G	0.93 (0.65,1.32)		1.46 (0.88,2.43)	
G/G	0.76 (0.49,1.19)		1.49 (0.77,2.86)	
rs10863399ref.=A/A		0.527		0.054
A/C	1.05 (0.75,1.48)		1.69 (1.04,2.73)	
C/C	1.83 (0.62,5.44)		0.51 (0.11,2.44)	
rs4903359ref.=A/A		0.078		0.644
A/G	0.71 (0.52,0.98)		0.99 (0.63,1.55)	
G/G	1.18 (0.53,2.6)		1.59 (0.59,4.34)	
rs3917187ref.=C/C		0.689		0.964
C/T	1.14 (0.82,1.59)		0.94 (0.59,1.5)	
T/T	1.18 (0.59,2.34)		0.92 (0.35,2.42)	
rs2284792ref.=A/A		0.508		0.997
A/G	1.21 (0.87,1.68)		1.01 (0.64,1.6)	
G/G	1.0066(0.5123,1.9778)		1.03 (0.39,2.75)	
rs2268626ref.=T/T		0.862		0.375
T/C	0.9925(0.7071,1.393)		1.25 (0.77,2.02)	
C/C	1.25 (0.54,2.88)		1.94 (0.66,5.72)	

Furthermore, A/A genotype of rs4803455 presented to be protective against severe asthma development in comparison with C/C genotype. Multiple SNPs were significantly associated with asthma severity. Both C/T and T/T of rs2009112 were associated with an increased severity of asthma in comparison with C/C genotype, like C/T in rs2796821, A/G in rs2796822, A/C in rs10863399. In contrast, in the analysis of the risk of asthma diagnosis, only the C/C genotype in rs10779329 was associated with a significantly lower risk of disease and rs4903359 A/G in comparison with most common genotypes.

To assess the predictive abilities of studied SNPs, we have developed benchmark predictive models for both asthma diagnosis (asthma vs. healthy) and severity (severe vs. non-severe, as defined in the “Methods” section).

As shown in **Table 5**, out of the total number of 49 features, 23 remained after MRMR feature selection for modeling of asthma diagnosis prediction. For asthma severity, an application of prediction MRMR feature selection allowed to reduce the dimensionality of the dataset to 17 features.

**Table 5:** Performance metrics of developed final data-mining models.

Scenario	Included potential predictors	Set	Accur acy (95% CI)	Sensiti vity	Specifi city	Positiv e Predict ive Value	Negati ve Predict ive Value	AUC ROC (95% CI)
TGF+Clinical forasthmadiagnosis	age, sex, height, weight, BMI, allergy, rs8109627, rs8179181, rs4803455, rs1800469, rs10495098, rs17047703, rs17558745, rs2799085, rs2009112, rs10482751, rs2027567, rs10779329, rs2796821, rs2796822, rs2798631, rs10863399, rs4903359, rs3917187, rs2284792, rs2268626, macarrier_rs8109627, macarrier_rs8179181, macarrier_rs4803455, macarrier_rs1800469, macarrier_rs10495098, macarrier_rs17047703, macarrier_rs17558745, macarrier_rs2799085, macarrier_rs2009112, macarrier_rs10482751, macarrier_rs2027567, macarrier_rs10779329, macarrier_rs2796821, macarrier_rs2796822, macarrier_rs2798631, macarrier_rs10863399, macarrier_rs4903359, macarrier_rs3917187, macarrier_rs2284792, macarrier_rs2268626, FEV1, FVC, FEV1doFVC	Training	100%(99.3%-100%)	100%	100%	100%	100%	1
		Validat ion	81.5%(73.8%- 87.8%)	88.4%	73.8%	79.2%	84.9%	0.87(0.81-0.93)
Only Clinical for asthma diagnosis	age, sex, height, weight, BMI, allergy, FEV1, FVC, FEV1doFVC	Training	95.8%(93.7%-97.3%)	97.8%	93.5%	94.4%	97.5%	0.99(0.99-1.00)
		Validation	73.8%(65.4%-81.1%)	73.9%	73.8%	76.1%	71.4%	0.80(0.72-0.88)
MRMR for asthma diagnosis	age, FEV1doFVC,allergy, FVC, macarrier_rs4903359, rs10779329, rs4803455,BMI, macarrier_rs10495098, FEV1, rs8109627,height, macarrier_rs2799085, macarrier_rs8179181, macarrier_rs2027567, rs17558745, rs2268626, macarrier_rs2009112, rs1800469, macarrier_rs2798631, sex, rs10863399, macarrier_rs17047703	Training	99.8%(98.9%-100%)	99.6%	100%	100%	99.6%	~1
		Validat ion	76.2%(67.9%- 83.2%)	76.8%	75.4%	77.9%	74.2%	0.85(0.79-0.92)
TGF+Clinical for severity prediction	age, sex, height,weight, BMI,allergy, rs8109627, rs8179181, rs4803455, rs1800469, rs10495098, rs17047703, rs17558745, rs2799085, rs2009112, rs10482751, rs2027567, rs10779329, rs2796821, rs2796822, rs2798631, rs10863399, rs4903359, rs3917187, rs2284792, rs2268626, macarrier_rs8109627, macarrier_rs8179181, macarrier_rs4803455, macarrier_rs1800469, macarrier_rs10495098, macarrier_rs17047703, macarrier_rs17558745, macarrier_rs2799085, macarrier_rs2009112, macarrier_rs10482751, macarrier_rs2027567, macarrier_rs10779329, macarrier_rs2796821, macarrier_rs2796822, macarrier_rs2798631, macarrier_rs10863399, macarrier_rs4903359, macarrier_rs3917187, macarrier_rs2284792, macarrier_rs2268626, FEV1, FVC, FEV1doFVC, Control test, Number of exacerbations, Smoking pack years, Age of diagnosis	Training	97.5%(94.8%-99.0%)	97.0%	97.8%	96.0%	98.3%	~1(0.99-1.0)
		Validation	75.4%(63.5%- 85.0%)	64.0%	81.8%	66.7%	80.0%	0.76(0.64-0.88)
Only Clinical for severity prediction	age, sex, height, weight, BMI, allergy, FEV1, FVC, FEV1doFVC, Control test, Number of exacerbations, Smoking pack years, Age of diagnosis	Training	83.7%(78.8%-87.9%)	65.0%	94.3%	86.7%	82.6%	0.91(0.87-0.94)
		Validat ion	72.5%(60.4%- 82.5%)	44.0%	88.6%	68.8%	73.6%	0.75(0.62-0.87)
MRMR for severity prediction	age, Number of exacerbations FEV1, rs2009112, Control test, rs17558745, FEV1doFVC, rs4803455, rs2268626, FVC, Smoking packyears, Age ofdiagnosis, rs8109627,height, macarrier_rs10779329, allergy, BMI	Training	86.6%(82.0%-90.4%)	76.0%	92.6%	85.4%	87.2%	0.95(0.93-0.97)
		Validat ion	73.9%(61.9%- 83.8%)	52.0%	86.4%	68.4%	76.0%	0.77(0.65-0.89)

According to results of DeLong’s test, regarding asthma diagnosis, the predictive Clinical+TGF model presented better diagnostic potential (AUC ROC) than Only Clinical model in both training (p=0.0009) and validation (p=0.0052). At the same time, the MRMR model was not worse than the Clinical+TGF model (p=0.3607 on the training set, p=0.1590 on the validation set), while it was better in comparison with the Only Clinical model (p=0.0010 on the training set, p=0.0235 on validation set).

**Figure 4:**
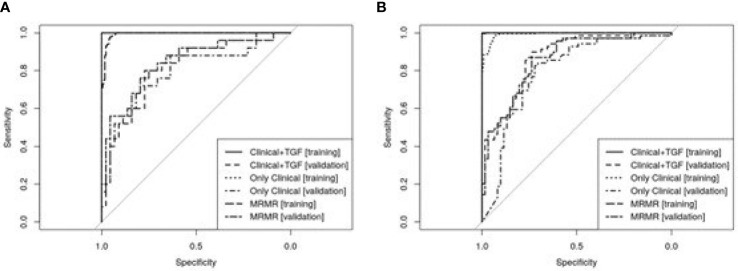
ROC curves showing predictive abilities of developed predictive models and their validation. Panel **(A)** presents models developed for asthma diagnosis prediction and panel **(B)** for asthma severity forecasting. A detailed description is included in the text.

Similar observations were not noted for asthma severity. Although the Clinical+TGF model was better than the Only Clinical model alone on the training set (p<0.0001), no difference in AUC ROC on the validation set was noted (p=0.7977), which indicated overfitting and lack of benefit from a SNP analysis. MRMR feature selection decreased predictive performance of models on the training set (p<0.0001) while the performance for validation remained similar (AUC ROC 0.77 vs. 0.76, p=0.8393). No statistically significant benefit was observed between Clinical+TGF, Only Clinical and MRMR models on validation sets. ROC curves were shown in **Figure 4**.

## Discussion

Despite the fact that allergy, which can be detected in around 90% of patients, a combination of genetic factors and other environmental determinants are responsible for an occurrence of the disease. From the clinical point of view, of all candidate gene groups of allergic diseases and asthma, those genes which are associated with the function of Th2 lymphocytes, epithelial cells and the lung function, bronchial remodeling and asthma severity are particularly important. This group includes TGF-β1, TGF-β2 and TGF-β3 genes (22–26).

Today, the importance and role of SNPs in the pathogenesis of asthma is widely discussed. It should be noted however that there are a lot of studies on this issue, conducted on populations of different character and different sizes. Not all results are always replicable, either. It should be pointed out that many studies have shown and confirmed the functional role of SNPs of TGF-β1, TGF-β2 and TGF-β3 genes in asthma (12, 17–19, 23, 24). It is important, from the point of view of basic and clinical sciences, to know how these SNPs influence signaling pathways regulated by the TGF-β gene in asthma.

In this paper we analyzed SNPs in TGFβ1, TGFβ2 and TGFβ3 genes. Their functions and effects on the expression of TGFβ1, TGFβ2 and TGFβ3 mRNA, as well as a new pool not yet studied in asthma, had been known before. We present a comprehensive analysis of 20 polymorphisms of TGF-β1, TGF-β2 and TGF-β3 genes as a predictor of the disease as well as its severity. SNPs were tested for both MAC differences between asthmatic patients and healthy controls as well as between patients with non-severe and severe asthma. There were no statistically significant differences regarding any of studied MACs of SNPs in TGF-β1, TGF-β2 and TGF-β3 genes between the asthmatics and healthy subjects. However, rs2009112 [1.85 (1.11, 3.1) p 0.016], rs2796821 [1.72 (1.1.2.69) p 0.017] and rs2796822 [1.71 (1.07,2.71) p 0.022] in the TGF-β2 gene between patients with non-severe and severe asthma appear to stand out. For the whole genotypes of this rs2009112 ref. = C/C [C/T 1.83 (1.06,3.16) and T/T 1.9 (1.02,3.54)] no significant differences (p = 0.056) were found between severe and non-severe asthma patients. In contrast, very strong statistically significant differences were observed for rs2796821 ref. = C/C [C/T 1.93 (1.22,3.05) and T/T 0.4 (0.09.1.88) p = 0.004] and rs2796822 ref. = A/A [A/G 2.02 (1.25, 3.28) and G/G 0.87 (0.41,1.85) p = 0.004]. The specific SNP may be more commonly presented in patients with asthma (increased risk of severe asthma), and it was higher by 93% for heterozygous forms of rs2796821 and by 102% for the heterozygote forms of rs2796822. A statistical analysis of single SNPs, particularly on selected genes, as shown in the results, provides incomplete knowledge on the role of SNPs in the development of asthma, as well as in its more severe forms. In our study, we did not confirm the role of several SNPs in the TGF-β1 gene, but we discovered a new functional significance of other SNPs in the TGF-β2 gene (6, 10–13, 16, 17). In this part of the work, genotyping of 20 in TGF-β1, TGF-β2 and TGF-β3 genes allowed to discover two new SNPs that increase the risk of asthma (rs10779329 and rs4903359). To confirm these analyzes, it is worth investigating in the future the expression of TGF/Smad signaling pathway on cell models, and in particular, to determine the levels of Smad2/3 and Smad4, due to the fact that these proteins play a special role in stimulating the synthesis of fibronectin, proteoglycans, type I and III collagen and the intensification of eosinophil chemotaxis after allergen exposure in bronchi of asthmatics (16–19).

Considering the fact that alleles occur in SNP with different frequency in different populations as well as different results in the analysis of genotypes of the same SNPs using different molecular techniques, it should be concluded that the statistical analysis of single SNPs is of low molecular and clinical importance in the development of chronic inflammatory respiratory diseases, such as asthma.

In the light of the above, in the subsequent part of the study, we tested whether the analysis of selected SNPs could increase the predictive potential of well-known clinical factors in terms of asthma diagnosis and severity prediction. By splitting the dataset (hold-out validation), performing hyperparameter optimization and analysis of ROC curves we followed the golden standard of predictive model development. To further check whether selection of particular SNPs and clinical features could counteract overfitting the MRMR algorithm was used for dimensionality reduction. In the results section we showed that not all clinical and genomic features are needed to develop overfitting-resilient model for asthma diagnosis prediction. Based on the metrics in hold-out validation, in our opinion, the MRMR model could be recommended for asthma diagnosis prediction in clinical settings. One can reuse our models for prediction using RDS files in **Supplementary Appendix** *via* predict function in R caret package. Anonymized data of an individual patient were added to the appendix to facilitate the reproducibility and further research.

However, few things have to discussed at this step. First, univariable analysis has shown limited independent association of particular SNPs with asthma diagnosis and severity. By application of C5.0 algorithm in this paper we were able to find more complex patterns that show the information gain, however, one has to acknowledge that derived model is the ensemble of decision tree, thus does not provide a simple explanation of the predictions. Additionally, Only Clinical model was not worse than MRMR model. Although, we applied standard data mining pipeline (with hyperparameter optimization and hold-out validation) the model requires further external validation. Furthermore, limitations inherited with technology could serve as a source of bias. Although the missing data rate was law, data imputation was required for predictive modelling. This was due to the specificity of subsequent experiments, impurities that can sediment the on SpectroCHIP and may interfere with signal detection, as well as the likelihood of DNA degradation in the obtained samples. Lastly, the results may be valid only for Polish population, which is quite homogeneous.

Nevertheless, in this study we show that analysis of selected SNPs in combination with selected clinical factors predicts the asthma diagnosis better than just clinical factors. Proven validity of MRMR model could implement preventative methods against asthma in particular groups of patients (asthma endotypes). Therefore, earlier identification of patients burdened with risk of more severe disease (carriers of specific SNPs in TGF-β1, TGF-β2 and TGF-β3 genes) is possible. This would facilitate faster implementation of intensive anti-inflammatory treatment (GCS, glucocorticoids) and prevent disease progression, exacerbations and bronchial remodeling (regulation of remodeling by TGF-β1, TGF-β2 and TGF-β3 genes).

## Summary

This work is the first in the Polish population to analyze the problem of the functional impact of 20 SNPs of TGF-β1, TGF-β2 and TGF-β3 genes on the risk of asthma. We have revealed new relationships between the occurrence of SNP rs10779329 and rs4903359 of the TGF-β2 gene and a statistically significantly increased risk of asthma. This observation is particularly important because the TGF-β gene affects eosinophil levels, bronchial hyperreactivity and bronchial obturation as well as clinical symptoms of asthma. The TGF-β1-3 gene complex is an important regulator of the immune response in asthma. We also proposed new predictive models which proven that analysis of selected SNPs in combination with selected clinical factors predicts the asthma diagnosis better than just clinical factors for asthma diagnosis prediction. This was not proved for asthma severity prediction. Good validation properties indicate that presented models may be of great clinical potential.

## Data Availability Statement

The original contributions presented in the study are included in the article/**Supplementary Material**. Further inquiries can be directed to the corresponding author.

## Ethics Statement

The studies involving human participants were reviewed and approved by Consent of the Bioethics Committee. The study was approved by the local ethics committee (Consent of Research Review Board at the Medical University of Lodz, Poland, No RNN/133/09/KE). At the commencement of the study, participants were invited to take part voluntarily. Before enrolment, a written informed consent was obtained from each patient. The patients/participants provided their written informed consent to participate in this study.

## Author Contributions

MP conceived of the presented idea. MP, KS, and MK developed the theory and performed the computations. KS and MK verified the analytical methods. PK and MP encouraged KS and MK to investigate SNP models in asthma and supervised the findings of this work. All authors discussed the results and contributed to the final manuscript.

## Funding

The study has been financed from the Polpharm Scientific Foundation, grant no. 16/XIV/2015, Poland.

## Conflict of Interest

The authors declare that the research was conducted in the absence of any commercial or financial relationships that could be construed as a potential conflict of interest.

## Publisher’s Note

All claims expressed in this article are solely those of the authors and do not necessarily represent those of their affiliated organizations, or those of the publisher, the editors and the reviewers. Any product that may be evaluated in this article, or claim that may be made by its manufacturer, is not guaranteed or endorsed by the publisher.

## Supplementary Material

Supplementary Table 1 Table showing the r-squared and p-values of pairwise linkage disequilibrium analysis.

Supplementary Figure 1 The figure presents an example of a mass spectrum for 20 analyzed SNPs in sample no. 524. The graph shows loci of all extend primers and possible alleles in different colors. The mass is expressed in daltons (Da).

Supplementary Figure 2 Mass spectra showing various alleles of rs2009112 polymorphism for selected samples: **(A)** The graph presenting the intensity of mass spectra for T homozygote, lack of signal for C allele; **(B)** The graph presenting the intensity of mass spectra for CT heterozygote; **(C)** The graph presenting the intensity of mass spectra for C homozygote, lack of signal for T allele.
