# Supplementary material for: Cytokine TGFβ Gene Polymorphism in Asthma: TGF-Related SNP Analysis Enhances the Prediction of Disease Diagnosis (A Case-Control Study With Multivariable Data-Mining Model Development)
Source: Front Immunol. 2022 Jun 14;13:746360. doi: 10.3389/fimmu.2022.746360 (PMC9238410; doi:10.3389/fimmu.2022.746360)
Supplement: Supplementary Table 1 — Table showing the r-squared and p-values of pairwise linkage disequilibrium analysis. [file Table_1.docx]

| **SNPs** | rs8109627 | rs8179181 | rs4803455 | rs1800469 | rs10495098 | rs17047703 | rs17558745 | rs2799085 | rs2009112 | rs10482751 | rs2027567 | rs10779329 | rs2796821 | rs2796822 | rs2798631 | rs10863399 | rs4903359 | rs3917187 | rs2284792 | rs2268626 |
| --- | --- | --- | --- | --- | --- | --- | --- | --- | --- | --- | --- | --- | --- | --- | --- | --- | --- | --- | --- | --- |
| rs8109627 | X | r2 = 0.1111; p < 0.0001 | r2 = 0.0165; p < 0.0001 | r2 =0.0003; p = 0.5438 | r2 < 0.001; p = 0.9824 | r2 = 0.004; p = 0.0229 | r2 = 0.0009; p = 0.2919 | r2 = 0.0014; p = 0.1745 | r2 = 0.0038; p = 0.0252 | r2 = 0.0031; p = 0.0444 | r2 = 0.0019; p = 0.1153 | r2 = 0.0055; p = 0.0072 | r2 =0.0001; p = 0.7785 | r2 < 0.001; p = 0.8506 | r2 = 0.0004; p = 0.4787 | r2 = 0.0066; p = 0.0034 | r2 = 0.0007; p = 0.3465 | r2 = 0.0009; p = 0.2685 | r2 =0.0001; p = 0.7627 | r2 = 0.0005; p = 0.4082 |
| rs8179181 |  | X | r2 = 0.0764; p < 0.0001 | r2 = 0.0192; p < 0.0001 | r2 =0.0003; p = 0.5113 | r2 < 0.001; p = 0.9378 | r2 =0.0001; p = 0.758 | r2 = 0.0033; p = 0.0372 | r2 = 0.0005; p = 0.4144 | r2 = 0.0022; p = 0.0869 | r2 = 0.006; p = 0.0052 | r2 = 0.0086; p = 0.0008 | r2 = 0.001; p = 0.2631 | r2 = 0.0038; p = 0.0262 | r2 = 0.0063; p = 0.0043 | r2 < 0.001; p = 0.91 | r2 = 0.0033; p = 0.0376 | r2 = 0.0044; p = 0.0163 | r2 = 0.0022; p = 0.0915 | r2 = 0.0032; p = 0.0417 |
| rs4803455 |  |  | X | r2 = 0.3707; p < 0.0001 | r2 < 0.001; p = 0.959 | r2 < 0.001; p = 0.873 | r2 =0.0003; p = 0.5178 | r2 < 0.001; p = 0.948 | r2 =0.0001; p = 0.6745 | r2 = 0.0009; p = 0.2784 | r2 < 0.001; p = 0.9434 | r2 < 0.001; p = 0.906 | r2 =0.0001; p = 0.7265 | r2 = 0.0005; p = 0.411 | r2 = 0.0035; p = 0.0318 | r2 = 0.0009; p = 0.282 | r2 = 0.0027; p = 0.0588 | r2 = 0.0005; p = 0.4023 | r2 = 0.0004; p = 0.4716 | r2 = 0.0026; p = 0.0634 |
| rs1800469 |  |  |  | X | r2 =0.0002; p = 0.5835 | r2 < 0.001; p = 0.8435 | r2 =0.0001; p = 0.7892 | r2 = 0.002; p = 0.1029 | r2 =0.0001; p = 0.6982 | r2 = 0.0029; p = 0.051 | r2 = 0.0046; p = 0.0142 | r2 = 0.0032; p = 0.0425 | r2 = 0.0018; p = 0.1259 | r2 = 0.0006; p = 0.3743 | r2 = 0.0034; p = 0.034 | r2 = 0.0076; p = 0.0016 | r2 = 0.0075; p = 0.0017 | r2 =0.0003; p = 0.5158 | r2 = 0.0006; p = 0.3882 | r2 = 0.0026; p = 0.0666 |
| rs10495098 |  |  |  |  | X | r2 = 0.3251; p < 0.0001 | r2 = 0.162; p < 0.0001 | r2 = 0.0154; p < 0.0001 | r2 = 0.0658; p < 0.0001 | r2 = 0.0649; p < 0.0001 | r2 = 0.0335; p < 0.0001 | r2 = 0.0188; p < 0.0001 | r2 = 0.001; p = 0.2611 | r2 = 0.0122; p =0.0001 | r2 = 0.0145; p < 0.0001 | r2 =0.0001; p = 0.7764 | r2 =0.0003; p = 0.5243 | r2 = 0.0035; p = 0.0316 | r2 = 0.0039; p = 0.0248 | r2 = 0.0014; p = 0.1794 |
| rs17047703 |  |  |  |  |  | X | r2 = 0.6793; p < 0.0001 | r2 = 0.0389; p < 0.0001 | r2 = 0.0007; p = 0.3399 | r2 = 0.0017; p = 0.134 | r2 =0.0001; p = 0.7271 | r2 =0.0003; p = 0.5282 | r2 = 0.0087; p = 0.0008 | r2 = 0.0008; p = 0.3041 | r2 = 0.0043; p = 0.0178 | r2 = 0.0016; p = 0.1447 | r2 = 0.0056; p = 0.007 | r2 = 0.0063; p = 0.004 | r2 = 0.0048; p = 0.012 | r2 = 0.0022; p = 0.0899 |
| rs17558745 |  |  |  |  |  |  | X | r2 = 0.0377; p < 0.0001 | r2 = 0.0076; p = 0.0016 | r2 = 0.0034; p = 0.0347 | r2 =0.0002; p = 0.5896 | r2 =0.0002; p = 0.5788 | r2 = 0.0276; p < 0.0001 | r2 = 0.0071; p = 0.0024 | r2 = 0.0155; p < 0.0001 | r2 = 0.0099; p =0.0003 | r2 = 0.0067; p = 0.0031 | r2 = 0.006; p = 0.0051 | r2 = 0.0036; p = 0.0296 | r2 = 0.0014; p = 0.1755 |
| rs2799085 |  |  |  |  |  |  |  | X | r2 = 0.3848; p < 0.0001 | r2 = 0.464; p < 0.0001 | r2 = 0.4035; p < 0.0001 | r2 = 0.02; p < 0.0001 | r2 = 0.0459; p < 0.0001 | r2 = 0.0546; p < 0.0001 | r2 = 0.0453; p < 0.0001 | r2 = 0.0184; p < 0.0001 | r2 = 0.0039; p = 0.0235 | r2 = 0.0032; p = 0.0412 | r2 = 0.0016; p = 0.1451 | r2 < 0.001; p = 0.8239 |
| rs2009112 |  |  |  |  |  |  |  |  | X | r2 = 0.4084; p < 0.0001 | r2 = 0.2713; p < 0.0001 | r2 = 0.0813; p < 0.0001 | r2 = 0.0272; p < 0.0001 | r2 = 0.0528; p < 0.0001 | r2 = 0.0365; p < 0.0001 | r2 = 0.002; p = 0.1076 | r2 = 0.0015; p = 0.1683 | r2 = 0.0038; p = 0.0257 | r2 = 0.0012; p = 0.2109 | r2 =0.0003; p = 0.5439 |
| rs10482751 |  |  |  |  |  |  |  |  |  | X | r2 = 0.6907; p < 0.0001 | r2 = 0.0997; p < 0.0001 | r2 = 0.0117; p =0.0001 | r2 = 0.056; p < 0.0001 | r2 = 0.0281; p < 0.0001 | r2 = 0.0045; p = 0.016 | r2 = 0.0045; p = 0.0158 | r2 = 0.002; p = 0.106 | r2 =0.0002; p = 0.5796 | r2 =0.0001; p = 0.7457 |
| rs2027567 |  |  |  |  |  |  |  |  |  |  | X | r2 = 0.0485; p < 0.0001 | r2 = 0.004; p = 0.022 | r2 = 0.0356; p < 0.0001 | r2 = 0.0201; p < 0.0001 | r2 = 0.0019; p = 0.1139 | r2 = 0.0027; p = 0.0625 | r2 = 0.0013; p = 0.1887 | r2 =0.0001; p = 0.6771 | r2 =0.0002; p = 0.5702 |
| rs10779329 |  |  |  |  |  |  |  |  |  |  |  | X | r2 = 0.0041; p = 0.0207 | r2 = 0.3435; p < 0.0001 | r2 = 0.2174; p < 0.0001 | r2 = 0.0371; p < 0.0001 | r2 = 0.0018; p = 0.1269 | r2 = 0.0005; p = 0.4364 | r2 = 0.0016; p = 0.1427 | r2 =0.0001; p = 0.6866 |
| rs2796821 |  |  |  |  |  |  |  |  |  |  |  |  | X | r2 = 0.4379; p < 0.0001 | r2 = 0.2902; p < 0.0001 | r2 = 0.6596; p < 0.0001 | r2 = 0.0031; p = 0.0439 | r2 =0.0001; p = 0.7759 | r2 < 0.001; p = 0.9412 | r2 = 0.0006; p = 0.3758 |
| rs2796822 |  |  |  |  |  |  |  |  |  |  |  |  |  | X | r2 = 0.6912; p < 0.0001 | r2 = 0.2804; p < 0.0001 | r2 = 0.0033; p = 0.0387 | r2 = 0.0009; p = 0.2817 | r2 =0.0002; p = 0.5958 | r2 < 0.001; p = 0.8829 |
| rs2798631 |  |  |  |  |  |  |  |  |  |  |  |  |  |  | X | r2 = 0.2499; p < 0.0001 | r2 = 0.0043; p = 0.0174 | r2 = 0.0014; p = 0.1803 | r2 = 0.001; p = 0.255 | r2 =0.0003; p = 0.5257 |
| rs10863399 |  |  |  |  |  |  |  |  |  |  |  |  |  |  |  | X | r2 = 0.0121; p =0.0001 | r2 = 0.0025; p = 0.0728 | r2 = 0.0008; p = 0.3192 | r2 < 0.001; p = 0.8228 |
| rs4903359 |  |  |  |  |  |  |  |  |  |  |  |  |  |  |  |  | X | r2 = 0.5881; p < 0.0001 | r2 = 0.5188; p < 0.0001 | r2 = 0.3629; p < 0.0001 |
| rs3917187 |  |  |  |  |  |  |  |  |  |  |  |  |  |  |  |  |  | X | r2 = 0.8426; p < 0.0001 | r2 = 0.5935; p < 0.0001 |
| rs2284792 |  |  |  |  |  |  |  |  |  |  |  |  |  |  |  |  |  |  | X | r2 = 0.711; p < 0.0001 |
| rs2268626 |  |  |  |  |  |  |  |  |  |  |  |  |  |  |  |  |  |  |  | X |

Supplementary table 1.

Supplementary Table 1. Table showing the r-squared and p-values of pairwise linkage disequilibrium analysis.

Title

Cytokine TGFβ gene polymorphism in asthma: TGF-related SNP analysis enhances the prediction of disease diagnosis (a case-control study with multivariable data-mining model development).

Panek Michał^1^, Stawiski Konrad^2^, Kuna Piotr^1^.

1 Medical University of Lodz, Department of Internal Medicine, Asthma and Allergy of The Medical University of Lodz, Lodz, PL

2 Medical University of Lodz, Department of Biostatistics and Translational Medicine of The Medical University of Lodz, Lodz, PL
