# Supplementary figures and images for: Cytokine TGFβ Gene Polymorphism in Asthma: TGF-Related SNP Analysis Enhances the Prediction of Disease Diagnosis (A Case-Control Study With Multivariable Data-Mining Model Development)
Source: Front Immunol. 2022 Jun 14;13:746360. doi: 10.3389/fimmu.2022.746360 (PMC9238410; doi:10.3389/fimmu.2022.746360)

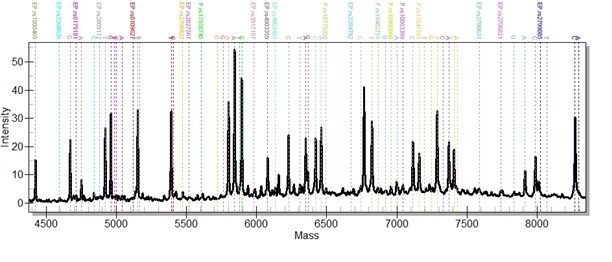

Supplement: Supplementary Figure 1 — The figure presents an example of a mass spectrum for 20 analyzed SNPs in sample no. 524. The graph shows loci of all extend primers and possible alleles in different colors. The mass is expressed in daltons (Da). [file Image_1.jpg]

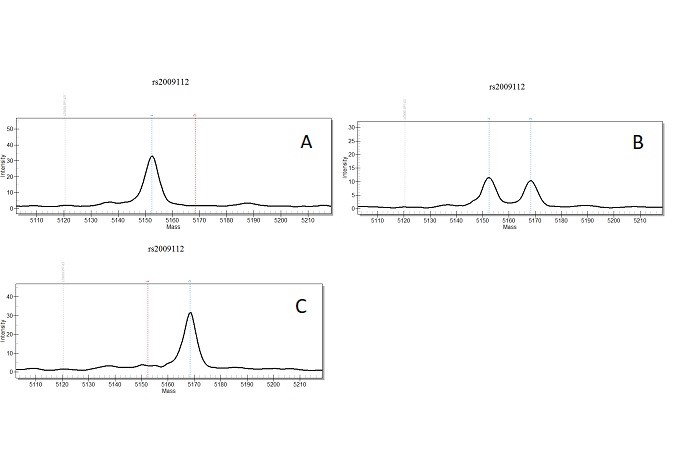

Supplement: Supplementary Figure 2 — Mass spectra showing various alleles of rs2009112 polymorphism for selected samples: (A) The graph presenting the intensity of mass spectra for T homozygote, lack of signal for C allele; (B) The graph presenting the intensity of mass spectra for CT heterozygote; (C) The graph presenting the intensity of mass spectra for C homozygote, lack of signal for T allele. [file Image_2.jpg]
