# Supplementary material for: Cytokine TGFβ Gene Polymorphism in Asthma: TGF-Related SNP Analysis Enhances the Prediction of Disease Diagnosis (A Case-Control Study With Multivariable Data-Mining Model Development)
Source: Front Immunol. 2022 Jun 14;13:746360. doi: 10.3389/fimmu.2022.746360 (PMC9238410; doi:10.3389/fimmu.2022.746360)
Supplement: Supplementary file 4 [file Table_2.docx]

Supplementary Table 2

| Analyzed groups | Asthmatic vs. control patients | | Severe vs. non-severe asthma | |
| --- | --- | --- | --- | --- |
| Studied SNPs | OR (95%CI) | P (LR-test) | OR (95%CI) | P (LR-test) |
| MAC of rs8109627 | 1.07 (0.78,1.47) | 0.657 | 0.78 (0.5,1.22) | 0.273 |
| MAC of rs8179181 | 0.89 (0.65,1.21) | 0.45 | 0.85 (0.55,1.33) | 0.476 |
| MAC of rs4803455 | 0.91 (0.65,1.29) | 0.612 | 0.68 (0.42,1.11) | 0.123 |
| MAC of rs1800469 | 1.09 (0.8,1.48) | 0.598 | 1.27 (0.81,1.98) | 0.295 |
| MAC of rs10495098 | 1.03 (0.74,1.43) | 0.88 | 1.02 (0.64,1.65) | 0.921 |
| MAC of rs17047703 | 0.95 (0.69,1.3) | 0.746 | 0.84 (0.53,1.32) | 0.442 |
| MAC of rs17558745 | 1.18 (0.87,1.61) | 0.287 | 0.67 (0.43,1.04) | 0.075 |
| MAC of rs2799085 | 0.97 (0.71,1.33) | 0.841 | 0.96 (0.61,1.5) | 0.852 |
| MAC of rs2009112 | 1.06 (0.76,1.49) | 0.729 | **1.85 (1.11,3.1)** | **0.016** |
| MAC of rs10482751 | 1.02 (0.75,1.39) | 0.907 | 1.01 (0.65,1.57) | 0.956 |
| MAC of rs2027567 | 1.03 (0.76,1.41) | 0.836 | 0.83 (0.54,1.3) | 0.417 |
| MAC of rs10779329 | 0.83 (0.61,1.14) | 0.249 | 0.93 (0.6,1.45) | 0.746 |
| MAC of rs2796821 | 1.2 (0.88,1.65) | 0.255 | **1.72 (1.1,2.69)** | **0.017** |
| MAC of rs2796822 | 0.96 (0.7,1.31) | 0.79 | **1.71 (1.07,2.71)** | **0.022** |
| MAC of rs2798631 | 0.88 (0.63,1.22) | 0.437 | 1.47 (0.91,2.38) | 0.113 |
| MAC of rs10863399 | 1.1 (0.79,1.53) | 0.589 | 1.54 (0.96,2.45) | 0.072 |
| MAC of rs4903359 | 0.74 (0.55,1.01) | 0.06 | 1.04 (0.67,1.61) | 0.863 |
| MAC of rs3917187 | 1.15 (0.84,1.57) | 0.39 | 0.94 (0.6,1.47) | 0.79 |
| MAC of rs2284792 | 1.18 (0.86,1.61) | 0.298 | 1.01 (0.65,1.58) | 0.948 |
| MAC of rs2268626 | 1.02 (0.73,1.41) | 0.914 | 1.32 (0.83,2.09) | 0.24 |
| rs8109627 ref.=T/T |  | 0.86 |  | 0.057 |
| T/C | 1.09 (0.79,1.51) |  | 0.67 (0.41,1.08) |  |
| C/C | 0.97 (0.49,1.94) |  | 1.99 (0.75,5.25) |  |
| rs8179181 ref.=G/G |  | 0.728 |  | 0.665 |
| G/A | 0.88 (0.63,1.21) |  | 0.89 (0.56,1.42) |  |
| A/A | 0.95 (0.53,1.71) |  | 0.69 (0.29,1.66) |  |
| rs4803455 ref.=C/C |  | 0.847 |  | 0.109 |
| C/A | 0.9 (0.62,1.3) |  | 0.78 (0.47,1.3) |  |
| A/A | 0.95 (0.61,1.46) |  | **0.51 (0.27,0.96)** |  |
| rs1800469 ref.=G/G |  | 0.251 |  | 0.547 |
| G/A | 1.08 (0.79,1.47) |  | 1.26 (0.81,1.98) |  |
| A/A | 726582.93 (0,Inf) |  | 2.02 (0.12,32.99) |  |
| rs10495098 ref.=G/G |  | 0.214 |  | 0.895 |
| G/T | 0.93 (0.65,1.32) |  | 1.07 (0.64,1.77) |  |
| T/T | 1.35 (0.86,2.13) |  | 0.93 (0.5,1.74) |  |
| rs17047703 ref.=C/C |  | 0.923 |  | 0.166 |
| C/A | 0.96 (0.69,1.34) |  | 0.93 (0.58,1.49) |  |
| A/A | 0.87 (0.41,1.88) |  | 0.28 (0.06,1.26) |  |
| rs17558745 ref.=C/C |  | 0.504 |  | 0.142 |
| C/T | 1.22 (0.88,1.69) |  | 0.71 (0.45,1.13) |  |
| T/T | 1.04 (0.57,1.9) |  | 0.47 (0.18,1.22) |  |
| rs2799085 ref.=C/C |  | 0.306 |  | 0.858 |
| C/A | 1.06 (0.75,1.48) |  | 0.9959 (0.6206,1.5982) |  |
| A/A | 0.74 (0.47,1.18) |  | 0.83 (0.41,1.69) |  |
| rs2009112 ref.=C/C |  | 0.76 |  | 0.056 |
| C/T | 1.02 (0.71,1.46) |  | **1.83 (1.06,3.16)** |  |
| T/T | 1.16 (0.76,1.77) |  | **1.9 (1.02,3.54)** |  |
| rs10482751 ref.=C/C |  | 0.454 |  | 0.966 |
| C/T | 1.09 (0.79,1.51) |  | 1.03 (0.65,1.63) |  |
| T/T | 0.78 (0.46,1.31) |  | 0.93 (0.42,2.06) |  |
| rs2027567 ref.=A/A |  | 0.866 |  | 0.667 |
| A/G | 1.06 (0.77,1.46) |  | 0.86 (0.54,1.36) |  |
| G/G | 0.9 (0.49,1.67) |  | 0.71 (0.28,1.81) |  |
| rs10779329 ref.=T/T |  | 0.091 |  | 0.886 |
| T/C | 0.92 (0.67,1.28) |  | 0.95 (0.6,1.51) |  |
| C/C | **0.53 (0.3,0.94)** |  | 0.8 (0.31,2.04) |  |
| rs2796821 ref.=C/C |  | 0.485 |  | 0.004 |
| C/T | 1.22 (0.88,1.69) |  | **1.93 (1.22,3.05)** |  |
| T/T | 1.04 (0.46,2.32) |  | 0.4 (0.09,1.88) |  |
| rs2796822 ref.=A/A |  | 0.26 |  | 0.004 |
| A/G | 1.05 (0.75,1.47) |  | **2.02 (1.25,3.28)** |  |
| G/G | 0.72 (0.46,1.15) |  | 0.87 (0.41,1.85) |  |
| rs2798631 ref.=A/A |  | 0.483 |  | 0.284 |
| A/G | 0.93 (0.65,1.32) |  | 1.46 (0.88,2.43) |  |
| G/G | 0.76 (0.49,1.19) |  | 1.49 (0.77,2.86) |  |
| rs10863399 ref.=A/A |  | 0.527 |  | 0.054 |
| A/C | 1.05 (0.75,1.48) |  | **1.69 (1.04,2.73)** |  |
| C/C | 1.83 (0.62,5.44) |  | 0.51 (0.11,2.44) |  |
| rs4903359 ref.=A/A |  | 0.078 |  | 0.644 |
| A/G | **0.71 (0.52,0.98)** |  | 0.99 (0.63,1.55) |  |
| G/G | 1.18 (0.53,2.6) |  | 1.59 (0.59,4.34) |  |
| rs3917187 ref.=C/C |  | 0.689 |  | 0.964 |
| C/T | 1.14 (0.82,1.59) |  | 0.94 (0.59,1.5) |  |
| T/T | 1.18 (0.59,2.34) |  | 0.92 (0.35,2.42) |  |
| rs2284792 ref.=A/A |  | 0.508 |  | 0.997 |
| A/G | 1.21 (0.87,1.68) |  | 1.01 (0.64,1.6) |  |
| G/G | 1.0066 (0.5123,1.9778) |  | 1.03 (0.39,2.75) |  |
| rs2268626 ref.=T/T |  | 0.862 |  | 0.375 |
| T/C | 0.9925 (0.7071,1.393) |  | 1.25 (0.77,2.02) |  |
| C/C | 1.25 (0.54,2.88) |  | 1.94 (0.66,5.72) |  |

Analysis of the frequency of alleles and genotypes of the tested SNPs in the group: healthy participants *vs* asthmatics, and non-severe *vs* severe asthma.
