# Supplementary material for: Cytokine TGFβ Gene Polymorphism in Asthma: TGF-Related SNP Analysis Enhances the Prediction of Disease Diagnosis (A Case-Control Study With Multivariable Data-Mining Model Development)
Source: Front Immunol. 2022 Jun 14;13:746360. doi: 10.3389/fimmu.2022.746360 (PMC9238410; doi:10.3389/fimmu.2022.746360)
Supplement: Supplementary file 5 [file Table_3.docx]

Supplementary Table 3

| Scenario | Included potential predictors | Set | Accuracy (95%CI) | Sensitivity | Specificity | Positive Predictive Value | Negative Predictive Value | AUC ROC (95%CI) |
| --- | --- | --- | --- | --- | --- | --- | --- | --- |
| TGF+Clinical for asthma diagnosis | age, sex, height, weight, BMI, allergy, rs8109627, rs8179181, rs4803455, rs1800469, rs10495098, rs17047703, rs17558745, rs2799085, rs2009112, rs10482751, rs2027567, rs10779329, rs2796821, rs2796822, rs2798631, rs10863399, rs4903359, rs3917187, rs2284792, rs2268626, macarrier_rs8109627, macarrier_rs8179181, macarrier_rs4803455, macarrier_rs1800469, macarrier_rs10495098, macarrier_rs17047703, macarrier_rs17558745, macarrier_rs2799085, macarrier_rs2009112, macarrier_rs10482751, macarrier_rs2027567, macarrier_rs10779329, macarrier_rs2796821, macarrier_rs2796822, macarrier_rs2798631, macarrier_rs10863399, macarrier_rs4903359, macarrier_rs3917187, macarrier_rs2284792, macarrier_rs2268626, FEV1, FVC, FEV1doFVC | Training | 100% (99.3%-100%) | 100% | 100% | 100% | 100% | 1 |
|  |  | Validation | 81.5% (73.8%-87.8%) | 88.4% | 73.8% | 79.2% | 84.9% | 0.87 (0.81-0.93) |
| Only Clinical for asthma diagnosis | age, sex, height, weight, BMI, allergy, FEV1, FVC, FEV1doFVC | Training | 95.8% (93.7%-97.3%) | 97.8% | 93.5% | 94.4% | 97.5% | 0.99 (0.99-1.00) |
|  |  | Validation | 73.8% (65.4%-81.1%) | 73.9% | 73.8% | 76.1% | 71.4% | 0.80 (0.72-0.88) |
| MRMR for asthma diagnosis | age, FEV1doFVC, allergy, FVC, macarrier_rs4903359, rs10779329, rs4803455, BMI, macarrier_rs10495098, FEV1, rs8109627, height, macarrier_rs2799085, macarrier_rs8179181, macarrier_rs2027567, rs17558745, rs2268626, macarrier_rs2009112, rs1800469, macarrier_rs2798631, sex, rs10863399, macarrier_rs17047703 | Training | 99.8% (98.9%-100%) | 99.6% | 100% | 100% | 99.6% | ~1 |
|  |  | Validation | 76.2% (67.9%-83.2%) | 76.8% | 75.4% | 77.9% | 74.2% | 0.85 (0.79-0.92) |
| TGF+Clinical for severity prediction | age, sex, height, weight, BMI, allergy, rs8109627, rs8179181, rs4803455, rs1800469, rs10495098, rs17047703, rs17558745, rs2799085, rs2009112, rs10482751, rs2027567, rs10779329, rs2796821, rs2796822, rs2798631, rs10863399, rs4903359, rs3917187, rs2284792, rs2268626, macarrier_rs8109627, macarrier_rs8179181, macarrier_rs4803455, macarrier_rs1800469, macarrier_rs10495098, macarrier_rs17047703, macarrier_rs17558745, macarrier_rs2799085, macarrier_rs2009112, macarrier_rs10482751, macarrier_rs2027567, macarrier_rs10779329, macarrier_rs2796821, macarrier_rs2796822, macarrier_rs2798631, macarrier_rs10863399, macarrier_rs4903359, macarrier_rs3917187, macarrier_rs2284792, macarrier_rs2268626, FEV1, FVC, FEV1doFVC, Control test, Number of exacerbations, Smoking pack years,  Age of diagnosis | Training | 97.5% (94.8%-99.0%) | 97.0% | 97.8% | 96.0% | 98.3% | ~1 (0.99-1.0) |
|  |  | Validation | 75.4% (63.5%-85.0%) | 64.0% | 81.8% | 66.7% | 80.0% | 0.76 (0.64-0.88) |
| Only Clinical for severity prediction | age, sex, height, weight, BMI, allergy, FEV1, FVC, FEV1doFVC, Control test, Number of exacerbations, Smoking pack years,  Age of diagnosis | Training | 83.7% (78.8%-87.9%) | 65.0% | 94.3% | 86.7% | 82.6% | 0.91 (0.87-0.94) |
|  |  | Validation | 72.5% (60.4%-82.5%) | 44.0% | 88.6% | 68.8% | 73.6% | 0.75 (0.62-0.87) |
| MRMR for severity prediction | age,  Number of exacerbations FEV1, rs2009112, Control test, rs17558745, FEV1doFVC, rs4803455, rs2268626, FVC,  Smoking pack years,  Age of diagnosis, rs8109627, height, macarrier_rs10779329, allergy, BMI | Training | 86.6% (82.0%-90.4%) | 76.0% | 92.6% | 85.4% | 87.2% | 0.95 (0.93-0.97) |
|  |  | Validation | 73.9% (61.9%-83.8%) | 52.0% | 86.4% | 68.4% | 76.0% | 0.77 (0.65-0.89) |

The presentation of architectures, features and performance of the statistical models used in the research.
